# Development of 3D Printed Bruch’s Membrane-Mimetic Substance for the Maturation of Retinal Pigment Epithelial Cells

**DOI:** 10.3390/ijms22031095

**Published:** 2021-01-22

**Authors:** Jongmin Kim, Ju Young Park, Jeong Sik Kong, Hyungseok Lee, Jae Yon Won, Dong Woo Cho

**Affiliations:** 1 Department of Mechanical Engineering, Pohang University of Science and Technology (POSTECH), Pohang 37673, Korea; mandarinbear@postech.ac.kr (J.K.); juyoung1489@postech.ac.kr (J.Y.P.); ahl@kangwon.ac.kr (H.L.); 2 School of Interdisciplinary Bioscience and Bioengineering, Pohang University of Science and Technology (POSTECH), Pohang 37673, Korea; urhere@postech.ac.kr; 3 Department of Mechanical and Biomedical Engineering, Kangwon National University, Chuncheon 24341, Korea; 4 Department of Ophthalmology and Visual Science, Eunpyeong St. Mary’s Hospital, The Catholic University of Korea, Seoul 03312, Korea; 5 Catholic Institute for Visual Science, College of Medicine, The Catholic University of Korea, Seoul 14662, Korea; 6 Institute of Convergence Science, Yonsei University, Seoul 03722, Korea

**Keywords:** retinal pigment epithelium, RPE maturation, tissue-specific bioink, in vitro RPE model, tissue-mimetic substrate

## Abstract

Retinal pigment epithelium (RPE) is a monolayer of the pigmented cells that lies on the thin extracellular matrix called Bruch’s membrane. This monolayer is the main component of the outer blood–retinal barrier (BRB), which plays a multifunctional role. Due to their crucial roles, the damage of this epithelium causes a wide range of diseases related to retinal degeneration including age-related macular degeneration, retinitis pigmentosa, and Stargardt disease. Unfortunately, there is presently no cure for these diseases. Clinically implantable RPE for humans is under development, and there is no practical examination platform for drug development. Here, we developed porcine Bruch’s membrane-derived bioink (BM-ECM). Compared to conventional laminin, the RPE cells on BM-ECM showed enhanced functionality of RPE. Furthermore, we developed the Bruch’s membrane-mimetic substrate (BMS) via the integration of BM-ECM and 3D printing technology, which revealed structure and extracellular matrix components similar to those of natural Bruch’s membrane. The developed BMS facilitated the appropriate functions of RPE, including barrier and clearance functions, the secretion of anti-angiogenic growth factors, and enzyme formation for phototransduction. Moreover, it could be used as a basement frame for RPE transplantation. We established BMS using 3D printing technology to grow RPE cells with functions that could be used for an in vitro model and RPE transplantation.

## 1. Introduction

The retinal pigment epithelium (RPE) is a monolayer of polarized and pigmented cells located between the retina and choroid of the eye. Lying on a thin extracellular matrix called Bruch’s membrane (BM), the RPE plays multifunctional roles, including light absorption, supporting visual function, clearance function through the phagocytosis of the photoreceptor outer segments, and secreting anti-angiogenic factors to prevent neovascularization in the retina [1,2,3,4]. In addition, the RPE forms a tight junction to control molecular transportation, which is called the outer blood–retinal barrier (BRB), together with the BM [5,6,7].

Due to their crucial roles, the loss of this epithelium disrupts the outer BRB and breaks down the metabolic circuitry of the ocular system, which causes a wide range of diseases related to retinal degeneration such as age-related macular degeneration (AMD), retinitis pigmentosa (RP), and Stargardt disease (STGD) [8,9,10,11]. Although these diseases are leading causes of blindness worldwide, they do not yet have an effective treatment. Anti-VEGF is a treatment option for patients; however, it only prevents the disease’s progression [12,13]

Transplantation is a promising treatment to replenish damaged RPE [14,15,16]. Several strategies have been used for RPE replacement so far, including injection of RPE cells and implantation of a sheet or patch containing an RPE monolayer [17,18,19]. For the treatment of a patient with dysfunctional RPE, the development of their natural functions is essential. Currently, the allogeneic fetal RPE or autologous RPE is transplanted into AMD patients; however, the RPE cells of these methods are immunogenic or require labor-invasive harvesting procedures [20,21]. Human induced pluripotent stem cells (hiPSc) have recently been used to develop the RPE monolayer and were implanted to into the patients with neovascular AMD; however, their best-corrected visual acuity was nearly unchanged [22]. Since AMD affects both the RPE and BM, transplantation could not form a proper microenvironment [23].

Several molecules, including small molecules and antibodies, are also under development to cure damaged RPE in the pharmaceutical industries [24]. During the development of drugs, cells are cultured on porous synthetic membranes to evaluate the products’ performance [25]. Alternative artificial substrates such as an electrospinning membrane that recapitulates the BM structure were recently used to fabricate a thin scaffold for RPE cell cultivation [26,27]. The RPE monolayer grown on these membranes showed improvement in function compared to conventional cultivation platforms.

Both transplantation and in vitro models require RPE with natural functions. Most of the cultured RPE was previously cultured on a thin porous membrane to mimic its natural habitat structure. However, these kinds of cultivation platforms cannot mimic the microenvironment related to the extracellular matrix (ECM) components because it focuses on the structural environment [28,29]. Several molecules, including laminin, collagen, and fibronectin, were used to mimic the RPE-specific microenvironment; however, individual or combinations of ECM components are insufficient to simulate the complex ECM microenvironment of RPE, as it lies on a BM composed of various kinds of molecules [30,31,32].

In this study, we developed Bruch’s membrane-derived ECM bioink (BM-ECM). The developed bioink facilitated RPE cells to show their natural functions, including barrier and clearance functions, the polarized secretion of anti-angiogenic factor, and enzyme formation for phototransduction. Finally, the integration of 3D printing technology and BM-ECM was used to fabricate a Bruch’s membrane-mimetic substrate (BMS) that could provide an optimized structural and ECM environment for RPE cells. Compared with conventional Transwell, the RPE cells on BMS showed enhanced natural functions. Moreover, the versatility of the BMS was shown by subretinal and subcutaneous implantation in rats. Therefore, from experimental observations, the developed BMS could be helpful in RPE-based research and for further applications.

## 2. Results

### 2.1. Characterization of Bruch’s Membrane-Derived ECM

**Figure 1:**
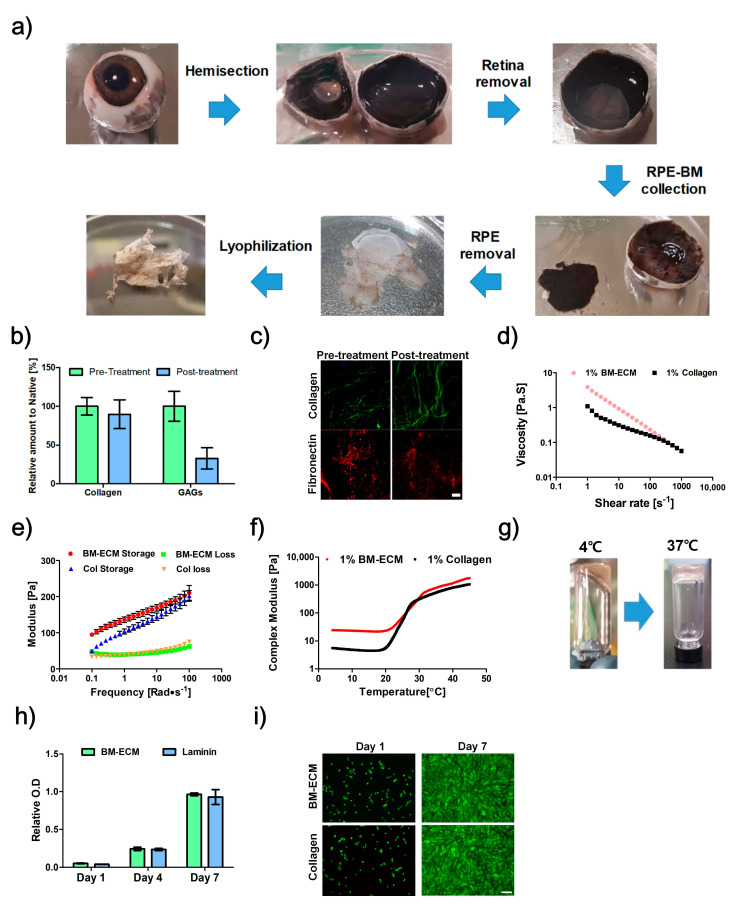
The development process of Bruch’s membrane-derived extracellular matrix bioink (BM-ECM). (**a**) Isolation procedure for BM. (**b**) Biochemical analysis of collagen and glycosaminoglycans (GAGs) for BM pre- and post-treatment with sodium dodecyl sulfate (SDS). (**c**) Immunofluorescence images of collagen type-1 and fibronectin for BM pre- and post-treatment with SDS. Rheological analysis results, (**d**) Viscosity, (**e**) storage modulus and loss modulus, and (**f**) complex modulus of BM-ECM and collagen in different conditions. (**g**) Sol-gel transition of 1% BM-ECM as a result of an increase in temperature. (**h**) Proliferation of ARPE-19 on non-coated, collagen, and BM-ECM-coated surfaces. (**i**) Live/Dead assay of ARPE-19 on non-coated, collagen, and BM-ECM-coated surfaces (Green: Live, Red: Dead). Scale bar: (**c**) 50 μm, (**i**) 200 μm. The error bars represent the standard deviation.

The BM-ECM was developed from porcine BM in a multistep process through sodium dodecyl sulfate (SDS) treatment, lyophilization, and pepsin digestion (Figure 1a). The biochemical components of products were determined and compared between pre- and post-treatment SDS; the results showed that 89.8% of collagen and 33% of glycosaminoglycans (GAGs) were conserved (Figure 1b). Immunofluorescence examination also confirmed conservation of collagen type-1 and fibronectin (Figure 1c).

The rheological characteristics of BM-ECM were determined in terms of viscosity and dynamic modulus. The viscosity of BM-ECM and collagen decreased as the shear rate increased, indicating a shear-thinning behavior (Figure 1d). The observed value of the storage modulus was greater than that of the loss modulus at 37 °C (Figure 1e). In the complex modulus, it rapidly increased when the temperature increased from 20 to 37 °C (Figure 1f). The thermal-sensitive crosslinking behavior was confirmed by performing a sol-gel transition test. The liquid state at 4 °C changed to a gel state at 37 °C (Figure 1g).

### 2.2. Proliferation Assay

The RPE cells were cultured on BM-ECM-coated Transwell and compared with those on laminin-coated Transwell. Similar proliferation was observed in both groups, and no dead cells were observed, which means that the developed BM-ECM is non-toxic (Figure 1h,i).

### 2.3. Barrier Function

**Figure 2:**
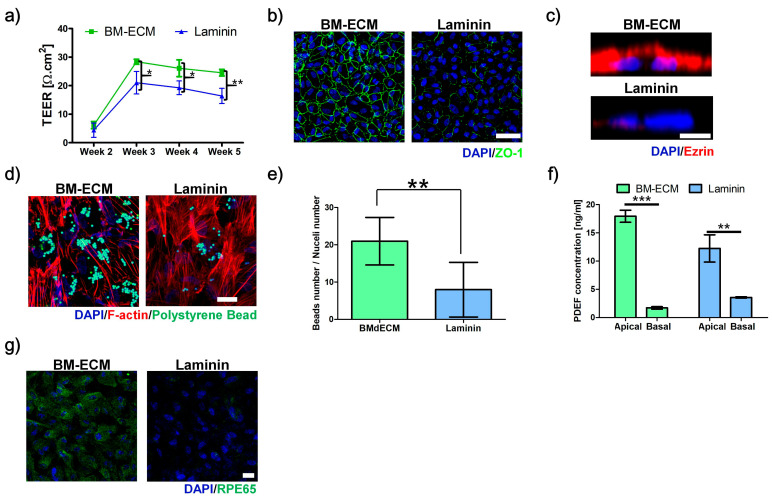
Effect of Bruch’s membrane-derived extracellular matrix bioink (BM-ECM) for retinal pigment epithelium (RPE) maturation. (**a**) Transepithelial electrical resistance (TEER) values at various times. (**b**) Expression of ZO-1 for tight junction, (**c**) Ezrin for microvilli formation, and (**d**) digested polystyrene beads for phagocytosis activity. (**e**) Ratios of digested beads numbers to nucleus numbers. (**f**) Secretion of pigment epithelium-derived factor (PEDF) in apical and basal medium for polarized PEDF secretion. (**g**) Expression of RPE65 for phototransduction in ARPE-19 cultured on BM-ECM- (BM-ECM) or laminin-coated Transwell (Laminin). Scale bar: (**b**) 50 μm, (**c**) 5 μm, (**d**), and (**g**) 20 μm. The error bars represent the standard deviation. The data were compared using Student’s t-test and differences were considered significant when * for *p* < 0.05, ** for *p* < 0.01, and *** for *p* < 0.001 (*n* = 3).

To evaluate the barrier function, the transepithelial electrical resistance (TEER) and expression of ZO-1 were compared. After two weeks, there were no significant differences in TEER values between BM-ECM and laminin-coated Transwell (Figure 2a). However, the TEER values of both RPE cells increased after three weeks with a greater value for BM-ECM-coated Transwell. The TEER value was nearly unchanged after three weeks, indicating that the RPE cells on BM-ECM showed a larger TEER value than those on laminin during the experiment. The formation of the tight junction was confirmed via expression of ZO-1. The RPE cells on BM-ECM-coated Transwell at three weeks showed a clear ZO-1 signal, while those on laminin indicated the incomplete formation of ZO-1 (Figure 2b).

### 2.4. Microvilli Formation

The microvilli formations were compared using immunostaining of ezrin (Figure 2c). The strong expression of ezrin was observed on the apical side of RPE cells cultured on BM-ECM-coated Transwell at three weeks. In contrast with BM-ECM-coated Transwell, the signal of ezrin was nearly unexpressed in RPE cells on laminin-coated Transwell.

### 2.5. Phagocytosis Ability

The function of phagocytosis was confirmed by the digestion of polystyrene beads (Figure 2d,e). The confocal microscope image showed that RPE cells on BM-ECM-coated Transwell had more active phagocytosis ability than that on laminin-coated Transwell, since greater numbers of digested polystyrene beads were observed in RPE cells on BM-ECM.

### 2.6. Polarized Secretion of PEDF

The polarized secretions of PEDF were compared using the measurement of PEDF concentration in media from apical and basal chambers. Both RPE cells on BM-ECM and laminin-coated Transwell showed the preference of secreting PEDF into the apical side than the basal side. However, the difference between apical and basal was more significant in RPE cells on BM-ECM than that on laminin (Figure 2f).

### 2.7. Expression of RPE65

To evaluate the function of phototransduction, the expression of RPE65—an enzyme for the visual cycle that converts all-trans-retinyl esters into 11-cis-retinol during phototransduction—in RPE cells was examined via immunocytochemistry (Figure 2g). The RPE cells on BM-ECM-coated Transwell exhibited strong expression of RPE65, while the signal was nearly undetectable in that on laminin.

### 2.8. Fabrication of 3D-Printed BMS

**Figure 3:**
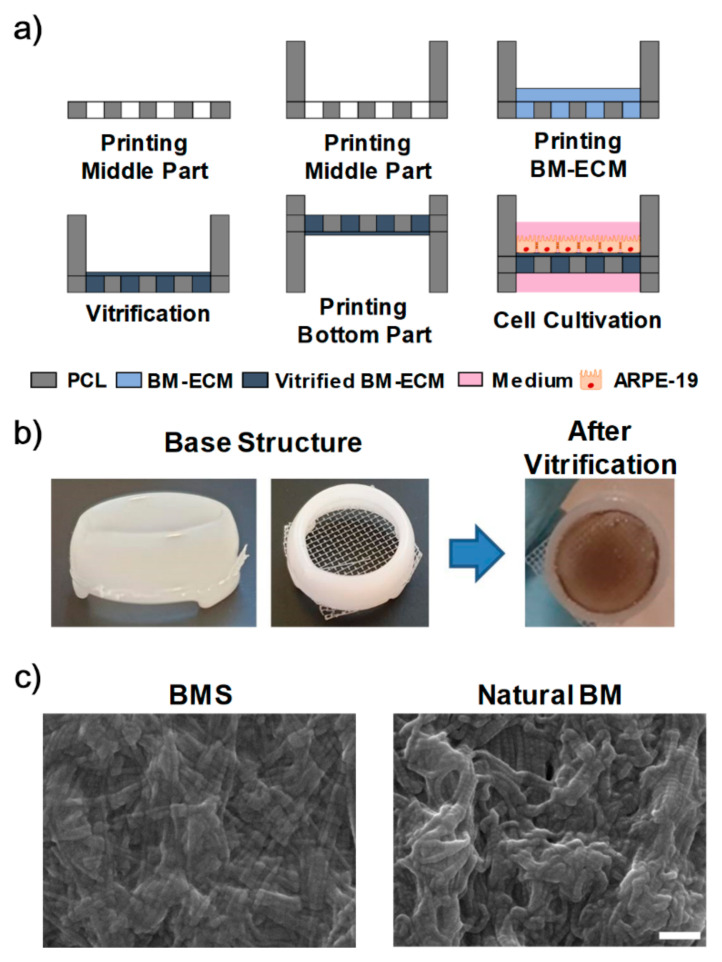
Development of Bruch’s membrane-mimetic substrate (BMS). (**a**) 3D printing process for BMS. (**b**) Photographs of fabricated products at two different stages. (**c**) SEM images of BMS and natural BM. Scale bars: 500 nm.

The BMS was fabricated with a combination of 3D printing technology and BM-ECM (Figure 3a,b). The fabricated BMS showed a fibrillar structure and ECM components as in the natural BM (Figure 3c, Figures S1 and S2), with similar TEER values as conventional Transwell, which indicated its feasibility (Figure S3).

### 2.9. 3D-Printed RPE Model

**Figure 4:**
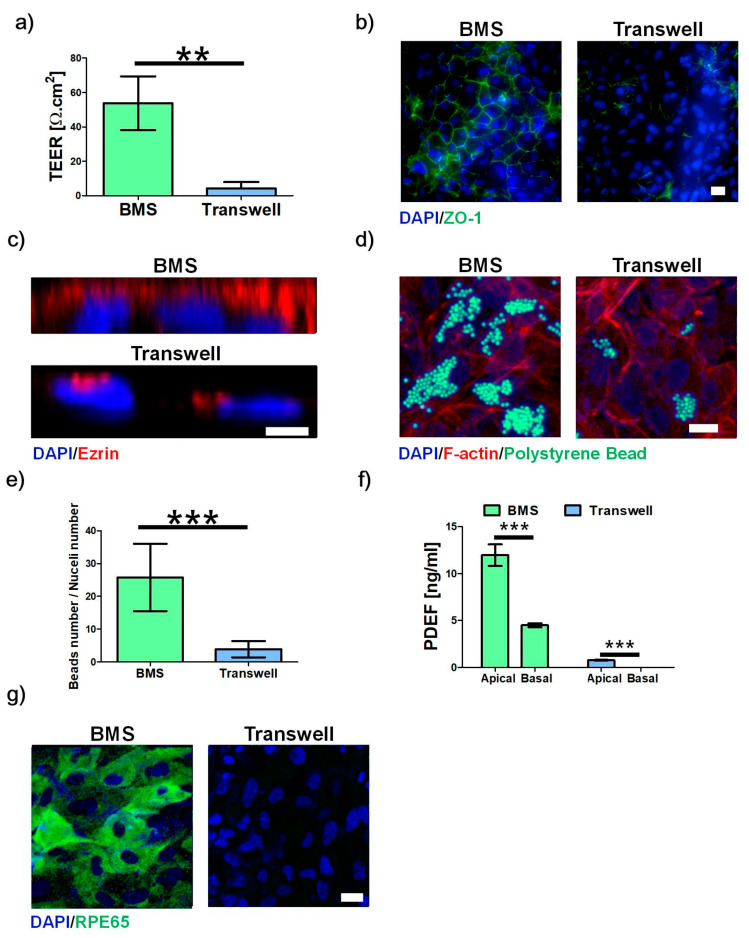
Effect of BMS on RPE maturation. (**a**) TEER value, (**b**) ZO-1 expression for tight junction, (**c**) ezrin expression for microvilli formation, (**d**) digested polystyrene beads for phagocytosis activity, (**e**) counted number of beads to nuclei ratios, (**f**) PEDF secretion for polarized secretion, and g) RPE65 formation for phototransduction in RPE cells on BMS or BM-ECM-coated Transwell (Transwell) at day 10. Scale bars: 20 μm (**a,c,g**), 5 μm (**b**). The error bars represent the standard deviation. The data were compared using Student’s t-test and differences were considered significant when ** for *p* < 0.01, and *** for *p* < 0.001 (*n* = 3).

The RPE cells were cultured on BMS and compared with those on Transwell. Regarding barrier function, the RPE cells on BMS showed a larger value for TEER and a stronger expression of ZO-1 than those on Transwell on day 10 (Figure 4a,b). Similarly, the enhanced clearance function (Figure 4c–e), polarized PEDF secretion (Figure 4f), and enzyme formation (Figure 4g) were observed in RPE cells on BMS.

### 2.10. Implantation of BMS

**Figure 5:**
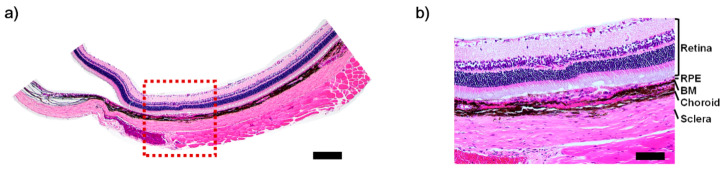
Histology image of a rat with subretinal implanted BMS. (**a**) H&E staining of the implanted BMS. (**b**) Magnification of subset (red box). Scale bars: (**a**) 500 μm; (**b**) 100 μm.

To confirm the ability of BMS as a transplantation platform, the fabricated BMS was implanted in the subcutaneous and subretinal region in rat. After one week of implantation, the rats were sacrificed and tissues were isolated. The BMS implanted in the subcutaneous region showed a well-preserved structure, indicating suitable mechanical properties for implantation (Figure S4). In the case of the subretinal implantation, we observed the stable attachment of BMS without any swelling or tearing via histology image (Figure 5a,b).

## 3. Discussion

RPE, together with its tight junctions forming the outer BRB, has a crucial role in the ocular system. It closely interacts with the photoreceptors on the apical side and with the BM and choroid on the basal. In conventional in vitro models, mimicking this interaction is difficult, since 2D cultivation only exposes their bodies to the culture medium in one part, which caused the aberrant functionalities [33,34]. Currently, most RPE cells have been cultured on porous membranes such as Transwell [35,36,37,38,39,40,41,42,43,44,45,46,47]. However, it only mimics the systemic environment by allowing culture medium in both apical and basal sides through the track-etched pores.

To overcome this limitation, several types of membrane—including both synthetic and natural materials—have been reported. Hamilton et al. cultured RPE cells and HUVECs on the amnion membrane. Although the authors showed an enhanced TEER value and tight junction formation, the fabricated membranes did not reveal natural functions, including phagocytosis, polarized secretion of PEDF, or RPE65 formation [6]. Thumann et al. used a commercial collagen membrane that showed enhanced cell attachment, phagocytosis, and the presence of RPE65 [48]. However, their barrier functions were not analyzed. Galloway et al. developed a collagen-coated fibroin membrane for hIPS-derived RPE; though, compared to laminin-coated Transwell, their functions were nearly unchanged [49]. The biodegradable polymer film was used for human fetal retinal pigment epithelial cell [50]. Although cultured cells secreted ECM into the polymer film, their barrier function was not analyzed. Recently, an electrospun nanofiber mesh (ENM) was introduced to mimic the porous mesh-like fibrillary structures [51,52,53]. Warnke et al. fabricated ENM with similar fibrillary structure with BM and cultured primary RPE cells [28]. However, compared with cover glass, the cell attachment, proliferation, tight junction formation, and presence of RPE65 were nearly unchanged.

Previous studies have revealed that mimicking the structural environment of BM is insufficient to maturate the function of RPE, since BM is also a source for ECM components. Therefore, providing similar structures or combinations of a few ECM materials cannot mimic the complex natural BM environment. Tissue-specific bioink and 3D-printing technology have been used in tissue engineering recently, including in vitro models [54,55,56]. The bioink developed for target organs not only demonstrated biocompatibility, but also contained optimized ECM components for cells [57,58]. Compared with conventional collagen bioink, the cells with tissue-specific bioinks showed enhanced differentiation into tissue-specific lineages and structure maturation. Moreover, the advanced 3D printing technology could simultaneously print multi-materials, including synthetic polymers and biomaterials, with high accuracy. The combination of tissue-specific bioink and high-accuracy multi-material printing systems facilitates the fabrication of in vitro models with a 3D biomimetic structure [59].

For the maturation of RPE functionalities, we aimed to develop the substrate with a similar structure and ECM compositions to that of natural BM. For this goal, we first successfully isolated BM and developed the BM-ECM from the porcine eye through the chemical treatment and confirmed the preservation of ECM components.

Then, the BM-ECM was analyzed in terms of rheology and cytotoxicity for 3D bio-printing. The BM-ECM showed shear-thinning behavior, thermal crosslinking, and larger storage modulus. In particular, the modulus of BM-ECM was larger than that for the collagen at the same concentration. This difference indicates that the modulus was not only affected by collagen, but also by the interactions of complex ECM components, which suggests that gelated BM-ECM had a more stable and enhanced substrate under dynamic conditions. The bioink also showed similar abilities of proliferation and non-toxicity compared with laminin. These results indicated that the developed BM-ECM is a suitable material for 3D bio-printing.

The ECM components of BM-ECM are major factors to critically influence the function of RPE [60,61,62]. Therefore, we compared the RPE cells cultured on BM-ECM-coated Transwell with those on laminin-coated Transwell in terms of barrier and clearance functions, polarized secretion of anti-angiogenic factor, and enzyme formation for phototransduction. We observed a larger TEER value and strong ZO-1 formations in RPE cells on BM-ECM-coated Transwell at three weeks. The RPE forms the outer BRB and controls the molecular transportation between choroid and retina. This barrier function prevents the free transportation of molecules through the gap in the plasma membrane. However, without tight junction formation, the RPE could not control molecular transportation, even when they form a confluent monolayer. This result indicates that the developed BM-ECM maturated RPE cells form the tight junction with an enhanced barrier function.

The clearance of the photoreceptor outer segment (POS) is a crucial function of RPE [63]. The microvilli on the apical side contact with photoreceptor cells to remove POS via phagocytosis and support its renewal. Dysfunction of POS clearance leads to the degeneration of photoreceptors as in the Royal College of Surgeons mutant rat [64]. Therefore, we compared the formation of microvilli and phagocytosis function, and confirmed the enhanced clearance ability in BM-ECM via clear linear signal of microvilli on the apical side and that it digested more polystyrene beads.

The RPE tends to secrete an anti-angiogenic factor called PEDF apically to prevent the aberrant growth of blood vessels into the sub-retinal space. We confirmed that RPE cells on both surfaces showed a larger concentration of PEDF on the apical side, whereas RPE cells on BM-ECM showed a larger concentration in apical and smaller concentration in basal than those on laminin. RPE65 is an enzyme related to the visual cycle. The mutation of this enzyme leads to severe blindness, such as RP. We demonstrated the formation of RPE65 in RPE on BM-ECM, whereas those on laminin did not reveal the formation of RPE65, confirming the enhanced function for anti-angiogenic factor secretion and potentially supporting visual cycle.

Finally, we developed the BMS using a 3D printing system and confirmed the enhancement of RPE functionalities. It is well known that RPE cells require long-term culturing for the formation of their natural functions. The conventional laminin-coated Transwell cultivation system requires between four weeks to six months to express the characteristics of RPE [40,65]. Turowski et al. cultured RPE cells on a porcine lens capsule for six weeks to express ZO-1, which expressed a stable TEER value [66]. Moreover, Shadforth et al. used silk fibroin as a basement membrane and cultured for 16 weeks to express ZO-1 and form RPE65 [67]. Given that the materials cultivation system reported in other studies were developed from non-BM-related tissues, they could not provide natural niches for RPE. In contrast to these other materials, the developed BMS exhibited a similar fibillary structure and ECM components from natural BM. These two characteristics induced the rapid maturation of RPE.

In addition, the fabricated BMS could also be used as a transplantation platform. By removing the upper and lower parts, its shape changed to a culture platform in flat form, which could be implanted in both subcutaneous and subretinal spaces. The implanted BMS preserved its structure without folding or tearing, even when the rats were under normal activity. These results indicated that the fabricated BMS could be used in both the RPE cultivation system and the transplantation platform. However, further development in the transplantation technique is required, since the implantation procedure might damage the outer retinal region as in the reported research related to the subretinal transplantation of patch [48,68,69]. Furthermore, it is expected to be useable in other organ systems by changing the organ-specific bioinks and cells [57].

In this study, we used ARPE-19, the RPE cell line, and showed the maturated function of RPE. However, the cell line has limitations in its function, including low TEER value and lack of basal membrane formation [43,70]. Recently, hiPSc-derived RPE cells were used for in vitro and in vivo study [71,72]. We expect that the developed BMS could be a candidate for a hiPSc-derived RPE cell cultivation system. Furthermore, we also expect that BMS can act as transplantation material since various kinds of decellularized xenograft, including bone and vessel, have been reported, and further studies are essential to fulfill the clinical requirements for transplantation such material preparation and good manufacturing practice (GMP) [73,74].

## 4. Conclusions

We have developed a BM-ECM bioink that can offer optimized ECM components for the maturation of RPE cells. Compared with conventional laminin-coated Transwell, the RPE cells on BM-ECM-coated Transwell showed enhanced functionality and performance. Furthermore, using 3D printing technology, we developed BMS that can provide a similar structure and ECM environment for RPE cells—allowing cells for maturation and maintenance of functionality—while also reducing the culture period. In addition, the developed cultivation system could be used as an experimental transplantation platform. Therefore, the BMS is an appropriate candidate to develop the functional RPE cell formation for both the in vitro model and transplantation.

## 5. Materials and Methods

### 5.1. Biochemical Characterization

Biochemical assays were performed to confirm the preservation of ECM components in BM-ECM. The concentrations of glycosaminoglycans (GAGs) were determined using 1,9-dimethyl methylene blue (Sigma-Aldrich, St. Louis, MO, USA), while chondroitin sulfate A (Sigma-Aldrich) was used to construct standard curves. For GAGs analysis, lyophilized native tissues and BM-ECM were digested with 125 μg/mL papain in 0.1 M sodium phosphate with 5 mM Na2-EDTA and 5 mM cysteine-HCl at pH 6.5 for 17 h at 60 °C. A papain solution without a sample for use as a diluent was also incubated in biochemical experiments. The GAGs in the tissue-digested solution had been stained with 1, 9-dimethyl methylene blue, following a 525 nm wavelength absorption samples read using a microplate spectrophotometer (Molecular Devices, San Jose, CA, USA). The collagen contents were quantified using a total collagen assay kit (Biovision, Milpitas, CA, USA). Samples were prepared using the manufacturer’s instructions. The samples’ absorbance was measured using a 540-nm-wavelength reader and quantified by comparison with a hydroxyproline standard curve.

### 5.2. Preparation of Bruch’s Membrane-Derived ECM

Samples of BM were isolated from porcine eyes, obtained from the local slaughterhouse within 1 h after slaughter, based on a previous procedure with slight modifications [75,76,77,78]. Briefly, the eyes were cleaned to detach the surrounding tissues, such as muscle, and were hemisected to remove the cornea and vitreous body. Then, the retina was peeled off using a disposable pipette. The remaining RPE–BM–choroid complex was collected and treated with 1% sodium dodecyl sulfate (SDS) solution with continuous stirring to remove the RPE and choroid layers. Then, the isolated BM was washed with distilled water and lyophilized and pepsin–acetic acid was used to digest the BM. In brief, 1 mg pepsin was added to 10 mg lyophilized BM in 3% acetic acid solution and left for 72 h at room temperature under constant stirring. The digested BM-ECM was neutralized using 0.1 N NaOH and 10× PBS.

### 5.3. Rheological Characterization

The rheological properties of BM-ECM were evaluated using the Discovery HR-1 rheometer system (TA instruments, New Castle, DE, USA) compared with collagen at the same concentration. The viscosity was measured using a steady shear sweep analysis method at 15 °C. The frequency-dependent storage (G′) and loss (G″) moduli were determined by conducting a dynamic frequency sweep analysis.

### 5.4. Cell Culture

The RPE cell (ARPE-19, CRL-2302, Lot number 70013110, Passage 20, American Type Culture Collection, Manassas, VA, USA) was cultured in DMEM with 10% FBS containing N1 supplement (Sigma-Aldrich), GlutaMax (Gibco), nonessential amino acids (NEAA, Gibco), taurine (Sigma-Aldrich), hydrocortisone (Sigma-Aldrich), and triiodo-thyronine (Sigma-Aldrich) on the permeable substrate (Transwell, 0.4-μm pore size, 6.5-mm diameter, Corning Coaster). Briefly, the Transwell was coated with 100 μg/mL laminin or BM-ECM for 2 h at 37 °C, followed by removing excess solution and rinsing with PBS twice [38,79]. After coating, 1.5 × 10^5^ cells/cm^2^ of RPE cells were plated on the BM-ECM or laminin-coated Transwell. The medium was added at the same height between the apical and basal chamber to prevent hydrostatic pressure while changing twice a week.

### 5.5. RPE Cell Proliferation Assay

The proliferation rate of RPE cells cultured on BM-ECM or a laminin-coated surface was determined using Cell Counting Kit-8 (CCK-8, Dojindo, Waltham, MA, USA). The 96-well plate was coated with 100 μg/mL of laminin or BM-ECM for 1 h at 37 °C, followed by washing with PBS twice. The 1.5 × 10^5^ cells/cm^2^ of RPE cells were seeded on the coated plate, and the cells were washed with PBS and incubated with CCK-8 solution for 1 h on days 1, 4, and 7. After incubation, the solution was collected and absorption at 450 nm was measured using a microplate spectrophotometer (Molecular Devices).

### 5.6. Live/Dead Assay

The viability values of the RPE cells were examined using a Live/Dead assay kit (Thermo Fisher Scientific, Waltham, MA, USA). Briefly, cultured RPE cells on the laminin or BM-ECM-coated plate were washed with PBS and treated with 2 μM calcein-AM and 4 μM ethidium homodimer-1 in culture medium for 10 min. After incubation, the RPE cells were washed with PBS and examined using a fluorescence microscope (Zeiss, Oberkochen, Germany).

### 5.7. Transepithelial Electrical Resistance

To evaluate the barrier function of RPE, the transepithelial electrical resistance (TEER) values were determined using an epithelial Volt-Ohm meter (EVOM2, World Precision Instruments) together with an STX2 chopstick electrode. In brief, the electrode was cleaned in 70% ethanol for 15 min and then washed with distilled water. The cleaned electrode was equilibrated in the culture medium for 10 min prior to use. Sides of the electrode were located in the apical and basal chambers, respectively, and the resistance value was measured. The TEER value was calculated by subtracting the blank resistance measured in a Transwell without a cell.

### 5.8. Immunostaining

Cultured RPE cells were fixed with 4% paraformaldehyde solution for 20 min at room temperature, permeabilized with 0.03% Triton-X in PBS for 7 min, and then washed with PBS. The samples were blocked with normal goat serum (Vector laboratory, San Francisco, CA, USA) and incubated with a 1:100 dilution of ZO-1 (Invitrogen, 33-9100, Carlsbad, CA, USA), RPE65 (Abcam, ab13826, Cambridge, MA, USA), and ezrin (Abcam, ab40839) antibody overnight at 4 °C. After incubation, the samples were washed with PBS with Tween-20 (PBST) three times followed by incubation with a 1:1000 dilution of secondary antibody conjugated with Alexa Fluor 488 or 594 (Invitrogen). After incubation of secondary antibody, the samples were washed with PBST three times and co-stained with a 1:1000 dilution of DAPI to label nuclei. The fluorescence image was taken using a confocal microscope (Zeiss).

### 5.9. Phagocytosis

To evaluate the functionality of the RPE, the ability of the phagocytosis was compared between RPE cells cultured on BM-ECM and laminin-coated Transwells. Each sample was incubated with 2 μm of fluorescence-labeled polystyrene bead (Invitrogen) at a concentration of 1 × 10^7^/mL for 16 h. After incubation, RPE cells were washed with PBS three times to remove undigested beads. The cells were fixed with 4% paraformaldehyde solution for 20 min and permeabilized with 0.03% Triton-X for 7 min at room temperature. After permeabilization, the samples were incubated with DAPI and TRITC phalloidin (Sigma-Aldrich). Imaging was carried out using a confocal microscope (Zeiss).

### 5.10. Enzyme-Linked Immunosorbent Assay (ELISA)

The concentration of PEDF in the medium of the apical and basal chamber was measured with a human PEDF ELISA (R&D) kit in accordance with the manufacturers’ instructions.

### 5.11. Fabrication of Bruch’s Membrane-Mimetic Substrate

The BMS was fabricated with a 3DXP multi-head 3D bioprinter (TnR Biofab). The polycaprolactone (PCL, molecular weight = 70,000–90,000 g/mol, Polysciences) was used as a basement structure for the BMS. The PCL was printed for the middle and upper parts of BMS at 90 °C and 300 kPa. Then, the BM-ECM was printed on the middle part at 10 °C and 50 kPa, and was incubated at 37 °C for gelation. The printed BMS was vitrified for three days to completely dry out. After vitrification, the bottom part was printed using PCL.

### 5.12. Cultivation of RPE Cells on BMS

RPE cells with a concentration of 1.5 × 10^5^ cells/cm^2^ were seeded on BMS with DMEM supplemented together with 10% FBS containing N1 supplement, GlutaMax, NEAA, taurine, hydrocortisone, and triiodo-thyronine. After the formation of the monolayer, their functions, including barrier and clearance functions, the secretion of anti-angiogenic factor, and enzyme formation, were analyzed.

### 5.13. Ethics Statement

All animal experiments were performed in strict accordance with a protocol approved by the Ethics Committee for Animal Experiments of St. Mary’s Hospital, the Catholic University of Korea (IACUC: CUMC-YEO20191702F). All surgical operations were performed under anesthesia with 2% inhaled isoflurane and intubated via the trachea with an 18-gauge intravenous catheter. All efforts were made to minimize suffering.

### 5.14. Animal Care

All rats used in this study were maintained in the specific-pathogen-free (SPF) area at St. Mary’s Hospital, the Catholic University of Korea. Rats were housed in a temperature-controlled container with a 12/12 h light/dark cycle, ad libitum access to food and water, and daily monitoring.

### 5.15. Animal Experiments

To confirm the attachment ability in tissue, the BMS was implanted in brown Norway rats (Orientbio). In brief, six rats of each group were anesthetized with inhaled isoflurane and topical (Proparacaine) anesthesia. Then, 2 mm × 5 mm and 1 mm × 1 mm of BMS specimens were implanted in the subcutaneous and subretinal spaces, respectively. The tissues were harvested one week after implantation [80,81], and rats were euthanatized using CO gas. The obtained tissues were fixed with 4% paraformaldehyde solution for one day, washed in ultrapure water, dehydrated in graded alcohol, and embedded in paraffin. Paraffin blocks were sectioned at 5-μm thickness following deparaffinization, rehydration, and H&E staining.

## Data Availability

Not applicable.

## Supplementary Materials

The following are available online at https://www.mdpi.com/1422-0067/22/3/1095/s1.
