# Supplementary material for: Development of 3D Printed Bruch’s Membrane-Mimetic Substance for the Maturation of Retinal Pigment Epithelial Cells
Source: Int J Mol Sci. 2021 Jan 22;22(3):1095. doi: 10.3390/ijms22031095 (PMC7865340; doi:10.3390/ijms22031095)
Supplement: Supplementary file 1 [file ijms-22-01095-s001.pdf]

Supporting Information

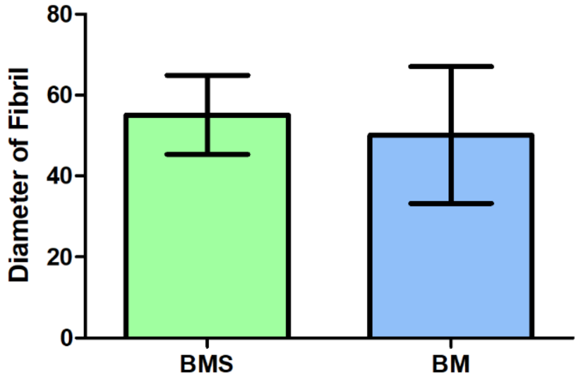

Figure S1. Diameter of fibril in Bruch's membrane mimetic substrate (BMS) and natural Bruch's membrane (BM). The diameter of each fibril was measured with ImageJ software. The error bars represent the standard deviation (n = 3).

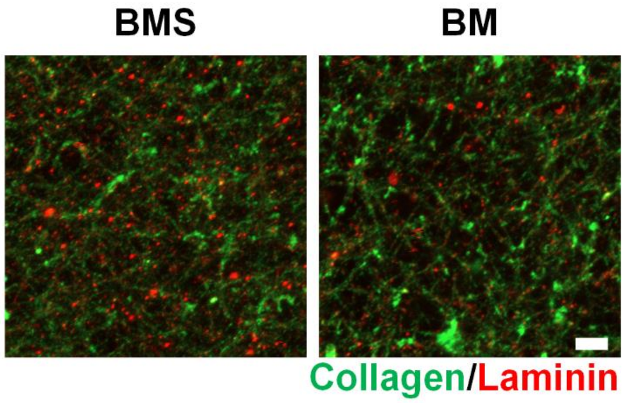

Figure S2. Immunostaining of collagen type 1 and laminin in Bruch's membrane mimetic substrate (BMS) and Bruch's membrane (BM). Scale bars: 5 μm

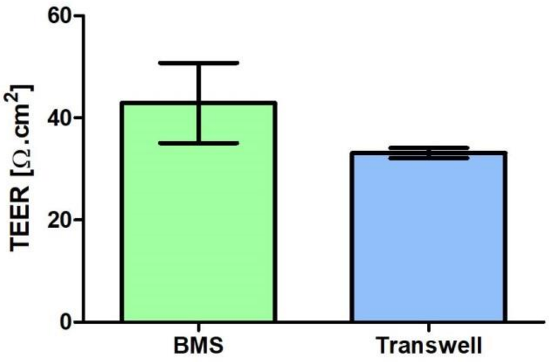

Figure S3. TEER value of the BMS and Transwell. The error bars represent the standard deviation (n = 3)

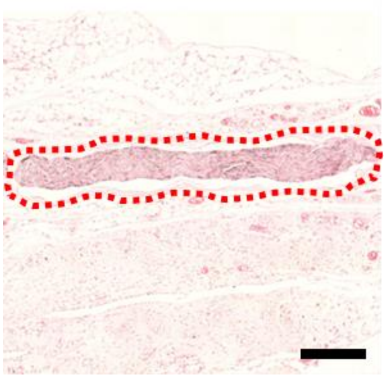

Figure S4. Histology of subcutaneous implanted BMS. Scale bars: 200 μm.
